# Removal of lithium from aqueous solutions by precipitation with sodium and choline alkanoate soaps†

**DOI:** 10.1039/d4gc05586a

**Published:** 2024-12-27

**Authors:** Stijn Raiguel, Dženita Avdibegović, Koen Binnemans

**Affiliations:** a KU Leuven, Department of Chemistry Celestijnenlaan 200F P.O. box 2404 B-3001 Leuven Belgium Koen.Binnemans@kuleuven.be

## Abstract

In order to comply with the expected tightening of discharge limits for lithium to surface waters, the lithium-ion battery industry will need access to methods to reduce the concentration of lithium in wastewater down to ppm levels. In this Communication, we discuss the possibility of using sodium and choline soaps as precipitating agents for lithium, comparing the two soap classes and probing the influence of the carbon chain length. It was found that lithium concentrations down to 10 ppm can be reached with sodium stearate, and down to 1 ppm with choline stearate, using a slight excess of the precipitating agent. However, in solutions containing sodium salts, sodium interferes with lithium removal, such that the equilibrium lithium concentration is proportional to the concentration of sodium in the feed. After precipitation, lithium could be recovered from the precipitate by dissolution in an ethanolic hydrogen chloride solution.

## Abstract 2

The possibilities and limitations of the use of sodium and choline soaps to remove lithium from wastewater were investigated. Very low residual concentrations are possible if conditions are met.

## Graphical abstract

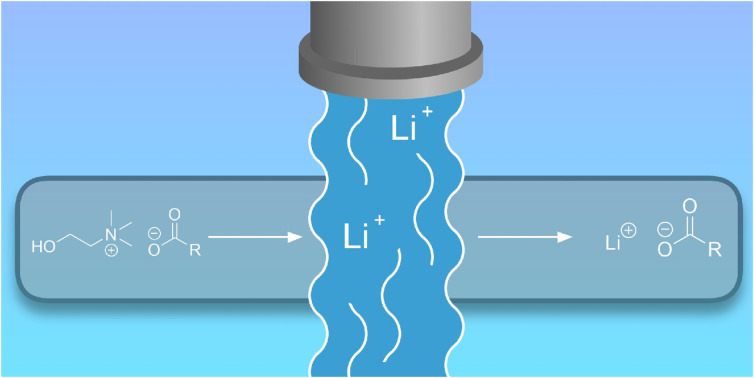

**Green foundation**

1. Lithium is a nephrotoxic and neurotoxic contaminant, that is becoming increasingly relevant as the production of cathode active materials for lithium-ion batteries continues to grow. Governmental agencies are thus moving towards implementing tight normative discharge limits. The industry is still struggling to achieve the expected limits, which are likely to be below 10 ppm. We report a method to precipitate lithium in the form of the lithium salt of a fatty acid (lithium soap).

2. Very low lithium concentrations, down to 1 ppm, can be achieved by precipitation using choline soaps. These concentrations are far lower than those obtained using state-of-the-art precipitation methods, such as precipitation as lithium phosphate or lithium carbonate.

3. The challenge is to deal with impurities such as sodium and higher valent metal ions that were found to interfere with lithium removal.

The rapid transition from internal combustion engines to electric vehicles has led to a spike in industrial activity related to the preparation and recycling of cathode active materials for lithium-ion batteries (LIBs). These production processes generate large volumes of industrial wastewater with high concentrations of lithium, usually in the form of lithium sulfate (Li_2_SO_4_).^1–3^ Lithium concentrations in excess of 5000 ppm are not uncommon.^3^ While aqueous streams are reused in LIB recycling processes, losses of lithium occur by way of bleeds and purges that are required to avoid build-up of impurities in the circulating aqueous streams. Similarly, refining of lithium from primary resources leads to the generation of industrial wastewater with elevated concentrations of lithium.

These industrial activities have led to increases in lithium concentrations in certain surface waters and groundwater reservoirs.^4^ Most published studies agree that elevated levels of lithium in water induce toxic effects in aquatic organisms, with concentrations well below 1 ppm being reported as harmful to certain aquatic organisms.^4,5^ Concentrations well in excess hereof have been measured in surface waters as a direct result of anthropogenic activity.^5^ Moreover, there are indications that elevated lithium concentrations in drinking water could have negative health effects on humans.^5^ The mechanisms of lithium toxicity are generally well-established.^4^ For these reasons, normative discharge limits are being defined for the lithium-ion battery industry. Values will differ from country to country, but it can be assumed that the future norms will be very strict, with values well below 100 ppm Li.

Various methods of lithium removal have been described in the literature in the context of lithium recovery from natural brines.^6^ These include membrane separations (such as nanofiltration and electrodialysis), ion exchange and solvent extraction. However, high CAPEX (for membrane processes) and OPEX (due to the generation of dilute streams, adsorbent degradation, and membrane fouling) render these techniques unsuitable for treatment of wastewater, since the amount of lithium recovered from wastewater is insufficient to justify the cost of its removal by expensive techniques. Adsorption is more cost-effective, but stripping and regeneration of the adsorbent yields very dilute solutions. As a result, the challenging lithium sequestration step is simply transferred to another solution.^7^ Precipitation is thus the preferred technique for treatment of waste waters.

The state-of-the-art method for precipitation of lithium from aqueous solutions is precipitation in the form of lithium carbonate (Li_2_CO_3_). However, this method is not suitable for bringing lithium concentrations down to ppm levels in industrial effluents because the solubility of Li_2_CO_3_ in water is too high. In pure water, the solubility of Li_2_CO_3_ is 12.9 g L^−1^ (5947 ppm Li) at 25 °C and 6.9 g L^−1^ (3298 ppm Li) at 100 °C.^8^ Alternatively, lithium can be precipitated as lithium phosphate (Li_3_PO_4_).^9–11^ Li_3_PO_4_ is 30 to 40 times less soluble in water than Li_2_CO_3_, as no more than 0.0394 g of Li_3_PO_4_ dissolves in 100 g of water at 18 °C.^12^ This corresponds to about 70 ppm of lithium, which is still often considered too high for discharge to surface waters. Lithium fluoride (LiF) has a similar solubility to Li_3_PO_4_ (0.027 g per 100 g water at 18 °C, *i.e.* 72 ppm Li), and fluoride precipitation introduces fluoride impurities in water.^13^

In this Communication, we demonstrate how lithium can be removed from aqueous solutions to very low final concentrations (in the ppm range) by precipitation of lithium in the form of salts of *n*-alkanoic acids (fatty acids). We also describe the relevant equilibria. Such lithium alkanoates (also called *lithium soaps*) have very low reported solubilities, with values as low as 0.010 g L^−1^ for lithium stearate at 25 °C.^8^ This corresponds to an equilibrium lithium concentration of approximately 2.4 ppm. To the best of our knowledge, removal of lithium from wastewaters by precipitation of lithium soaps has not been investigated yet, except for two patents that disclose the process of precipitating lithium as lithium stearate by addition of a solution of *in situ* generated sodium stearate to an aqueous solution of a lithium salt.^14,15^

Sodium alkanoates dissolve in water in micellar form at temperatures above their so-called *Krafft point*. The Krafft point is the minimum temperature at which a surfactant will form micelles, and it varies between room temperature and 71 °C for 12 to 18-carbon sodium alkanoates (Table 1). Below their Krafft point, the soaps behave as poorly soluble salts.^16^ Hence, lithium-bearing solutions must be heated to a temperature above the Krafft point for a metathesis reaction to take place, which leads to precipitation of lithium alkanoate salts (eqn (1)):1
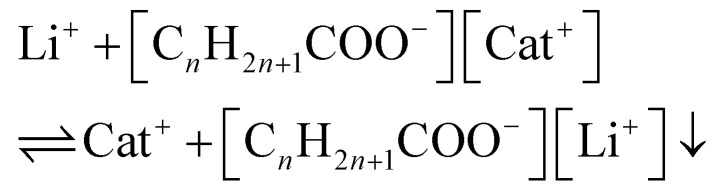


**Table 1:** Krafft points (°C) of the investigated alkanoate soaps^17^

Alkanoate	Sodium	Choline
Laurate (C12)	25	<0
Myristate (C14)	45	1
Palmitate (C16)	60	12
Stearate (C18)	71	40

The metathesis reaction is driven by the poor solubility of lithium soaps, which results from their Krafft points being above the boiling temperature of water.^18^ Choline soaps (Fig. 1) have significantly lower Krafft points than the corresponding sodium soaps.^17,19^ Choline laurate, myristate and palmitate have Krafft points below room temperature, while that of the choline stearate is only 40 °C (Table 1). The use of choline soaps, as opposed to sodium soaps, thus allows the precipitation process to be performed at significantly lower temperatures. Furthermore, choline and its soap are fully biodegradable and non-toxic, and hence compatible with wastewater treatment.^17^ Considering their potential advantages, four choline soaps were prepared and used to precipitate lithium from aqueous solutions. Their performance was compared to that of the corresponding sodium salts. In light of the importance of reducing the cost of the method for wastewater treatment, linear, even-carbon fatty acids were chosen for this study. Such fatty acids are readily available at low prices. The recovery of lithium from the precipitated lithium alkanoate salts was investigated as well. While choline salts are more expensive than sodium salts, choline alkanoates could be used for deep removal of trace amounts of lithium, after a rough precipitation using a conventional method.

**Fig. 1:**
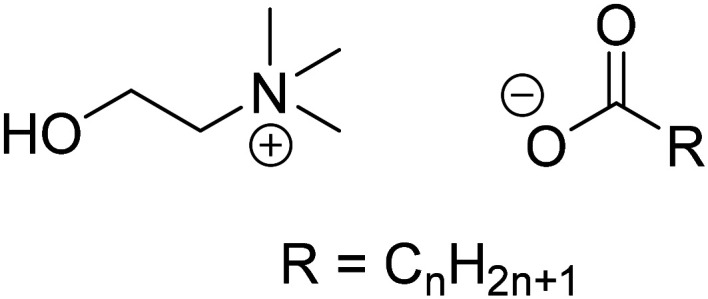
Structure of choline *n*-alkanoate soaps.

Sodium soaps were prepared by neutralization of fatty acids with sodium hydroxide, while choline palmitate and stearate were prepared by neutralization of fatty acids with choline hydroxide (detailed experimental procedures are given in the ESI, page S5†). A solution of lithium chloride containing 250 ppm of lithium was prepared, and 1.05 eq. of each soap was added to an aliquot of the solution. Samples were stirred and heated to 5 °C above the Krafft point of the added soap for 30 minutes, or stirred at ambient temperature (22 °C) if the Krafft point was below room temperature. Subsequently, the samples were allowed to cool and settle.

The residual lithium concentrations after precipitation are shown in Table 2. Interestingly, lower concentrations of lithium were found in samples treated with choline soaps compared to those treated with sodium soaps, in spite of the fact that reaction conditions were otherwise identical and that the same lithium-bearing product is formed by either class of soaps. XRD analysis of the residue obtained from the treatment of lithium chloride solution with choline stearate identified the precipitate as lithium stearate (see ESI, Fig. S1†). The sample was not fully crystalline, as evidenced by the presence of a broad scattering band typical of amorphous samples.

**Table 2:** Lithium concentrations in filtrate after treatment of a 250 ppm lithium (as LiCl) solution with 1.05 eq. of soap at 5 °C above the Krafft point, filtered at 22 °C

Alkanoate	[Li^+^] after treatment with Na soap, ppm	[Li^+^] after treatment with choline soap, ppm	[Li^+^] according to solubility of Li soap at 25 °C, ppm (ref. 6)
Laurate	59.9	n.d.	63.0
Myristate	11.9	n.d.	10.7
Palmitate	11.3	1.7	3.9
Stearate	10.4	1.3	2.4

To exclude that the difference was due to kinetic factors, aliquots of lithium chloride solution were treated with either sodium myristate or sodium stearate under identical conditions for 3 days as opposed to 30 minutes. No significant change in the lithium concentration was found for either sample: 13.2 *vs.* 11.9 ppm for the myristate soap and 9.7 *vs.* 10.4 ppm for the stearate soap.

The two classes of soaps differ in their Krafft point. The amount of available fatty acid in solution decreases sharply below the Krafft point. This could affect lithium soap precipitation in two distinct manners. On one hand, competitive precipitation of the poorly soluble sodium soap may result in a shift of the equilibrium concentration of lithium ions. On the other hand, a drastically reduced amount of fatty acid in solution may cause the equilibrium to freeze at the Krafft point, meaning that the residual lithium concentration would be determined by the solubility of the lithium soap at the Krafft point of the sodium soap.

To distinguish between these two mechanisms, a sample series was prepared with various molar ratios of tetrabutylammonium bromide added to sodium stearate. The initial lithium concentration was 100 ppm, present as the chloride salt. A progressive decrease of the Krafft point from 60 °C to 35 °C was observed as the amount of tetrabutylammonium bromide increased from 0.20 to 1.00 eq. If the equilibrium froze at the Krafft point, this would lead to a concurrent, gradual decrease in equilibrium lithium concentration, as the solubility of lithium stearate increases with temperature.^8^ However, this trend was not observed. Instead, the residual lithium concentration remained constant over the tested range, at 4.5 ± 0.5 ppm. These results are in line with the hypothesis of competitive sodium alkanoate precipitation. Further evidence hereof was obtained by varying the concentration of sodium chloride in the feed solution. Below the Krafft point of the system, the solubility of both soaps is entirely determined by their solubility products *K*_SP_ (eqn (2)):^16^2
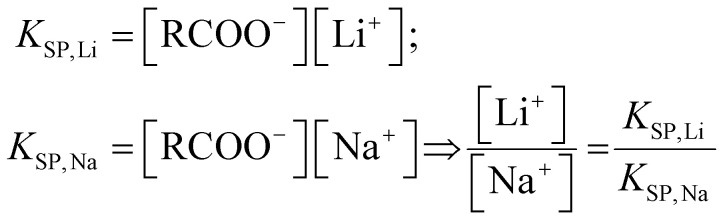


Increasing the concentration of sodium chloride should thus lead to a directly proportional increase in lithium concentration. This was found to be the case when adding 1.05 eq. of sodium palmitate to solutions containing 50 ppm of lithium and 0–150 ppm of sodium (Table 3). A constant lithium/sodium molar ratio of (44.7 ± 0.4) × 10^−3^ was measured, irrespective of the equilibrium sodium concentration. This implies that wastewaters with high concentrations of sodium cannot be treated effectively by precipitation with a fatty acid salt.

**Table 3:** Lithium and sodium concentrations in filtrate after treatment of a 50 ppm lithium (as LiCl) solution containing various amounts of NaCl with 1.05 eq. of lithium palmitate at 70 °C, filtered at 22 °C

[Na^+^] in feed, ppm	[Li^+^] after treatment with Na soap, ppm	[Na^+^] after treatment with Na soap, ppm	[Li^+^]/[Na^+^] molar ratio at equilibrium
0	2.5	181	0.046
45	3.0	224	0.044
90	3.5	258	0.045
150	4.3	320	0.044

In light of the competitive precipitation equilibrium, it seems reasonable to assume that filtering the sample above the Krafft point of the mixture would result in efficient precipitation of lithium in the presence of sodium, as sodium stearate is well-soluble above this point. An experiment similar to the aforementioned was performed, with the filtration being performed at 70 °C (Table 4) as opposed to room temperature. Afterwards, the solution was allowed to cool, and a second filtration was performed to remove the precipitated sodium palmitate. Surprisingly, similar concentrations of lithium were found in solution at equilibrium as for samples filtered at room temperature. This implies that relatively high concentrations of lithium must also be present above the Krafft point.

**Table 4:** Lithium and sodium concentrations in filtrate after treatment of a 50 ppm lithium (as LiCl) solution containing various amounts of NaCl with 1.05 eq. of lithium palmitate at 70 °C, filtered at 70 °C

[Na^+^] in feed, ppm	[Li^+^] after treatment with Na soap, ppm	[Na^+^] after treatment with Na soap, ppm	[Li^+^]/[Na^+^] molar ratio at equilibrium
0	2.3	192	0.039
75	2.9	238	0.041
150	3.6	285	0.042

In the older literature, “mixed micelles” have been invoked to explain a decrease in the Krafft temperature upon addition of salts to surfactant solutions. Mixed micelles with ions adsorbed to the micelle surface could solubilize otherwise unexpectedly large amounts of lithium.^20^ However, extensive ^7^Li NMR measurements demonstrate that Li^+^ ions remain largely free in solution above the Krafft point (see ESI, page S10†). This implies that the solubility product *K*_SP_ must remain met, as the Li^+^ ions are not sufficiently strongly adsorbed to the micelle surface for their activity in solution to drop significantly. On the other hand, it is well-established that inorganic salts can decrease the critical micelle concentration (CMC) of surfactants.^21^ As the CMC corresponds to the concentration of free surfactant molecules in equilibrium with micellar structures, an increase in the salt concentration would thus lead to a lower equilibrium concentration of alkanoate ions in solution. In turn, the solubility product predicts an increase in the dissolved Li^+^ concentration. Indeed, analysis of the reaction mixture above the Krafft point shows that larger concentrations of Li^+^ remains in solution above the Krafft point of the system, as the sodium chloride concentration increases (see ESI, page S9†). Thus, the sudden solubilization of sodium stearate above the Krafft point in fact leads to a further increase of the equilibrium lithium concentration, in spite of the cessation of the existence of the competitive precipitation equilibrium.

It is important to note that choline soaps will convert to sodium soaps in the presence of aqueous sodium ions, due to the poor solubility of sodium soaps at room temperature. Therefore, the advantages of choline soaps disappear when the feed solution contains sodium ions in addition to lithium ions. Addition of up to 3.0 eq. of choline chloride did not result in significant lowering of the Krafft point of 1.05 eq. sodium stearate or palmitate in 250 ppm lithium solutions. It must be noted that the calcium and magnesium soaps are also poorly soluble in water, so that these compounds will coprecipitate with lithium if calcium and magnesium salts are present in the lithium-containing solutions.

In addition to sodium and higher-valent ions, excess acid can also interfere with the precipitation reaction by protonating the alkanoate ions, rendering them unavailable for lithium soap formation. The minimal equilibrium pH for lithium precipitation was determined by titrating choline stearate (1.05 eq.) with hydrochloric acid (0–14 mM) in the presence of aqueous lithium solution (91 ppm as LiCl). It was found that the reaction between hydrochloric acid and choline stearate proceeds to completion, and the loss in lithium precipitation yield is equal to the mole fraction of hydrochloric acid added to the solution. The pH was buffered between 8.5 and 8.8 in samples with 0.2–0.8 eq. of hydrochloric acid added, indicating that the equilibrium pH must be above these values for maximal precipitation of lithium to occur (see ESI, Fig. S.6†).

The possibility of recovering lithium from the precipitated lithium soap was investigated using acid to displace lithium from its alkanoate salts. Aqueous hydrochloric acid (0.1 mol L^−1^, 22 °C) was not able to fully recover lithium from its stearate soap after 60 minutes of stirring. Only 15% was recovered in both duplicate samples at a solid-to-liquid ratio of 30 mg mL^−1^. By contrast, ethanolic hydrogen chloride proved exceedingly effective, fully dissolving lithium stearate within 10 minutes at a hydrogen chloride concentration of 0.025 mol L^−1^ and a solid-to-liquid ratio of 5 mg mL^−1^ (approx. 1.5 eq. of acid). Duplicate measurements afforded lithium recovery yields of 91% and 93%. As the sample was fully dissolved, it can be assumed that the deviation from 100% results from an impurity in the lithium stearate precipitate, such as absorbed moisture. The striking difference in performance of aqueous *versus* ethanolic hydrogen chloride can be attributed to the insolubility of stearic acid in water, leading to passivation of the lithium stearate particles by an inert layer of stearic acid.

In conclusion, both sodium and choline soaps are capable of removing lithium from aqueous solutions down to ppm levels, when added in a 5 mol% excess. Choline soaps can be used to obtain very low residual lithium concentrations of a few ppm and will react at lower temperatures than sodium soaps. These observations seem to result from the higher solubility and lower Krafft points of the choline soaps, compared to their respective sodium analogs. However, these advantages disappear if sodium is present in the feed solution, in which case the choline soaps convert to sodium soaps. For sodium-containing systems, the residual lithium concentration is proportional to the equilibrium sodium concentration. This renders the process ineffective for waste waters containing high concentrations of sodium. Recovery of lithium from the precipitated lithium soap is possible using an ethanolic hydrogen chloride solution. The possibility of reducing the concentration of lithium in wastewater to very low concentrations (ppm level) is of importance for the development of circular hydrometallurgical flowsheets for lithium.^22^ Solutions with a high lithium/sodium ratio, such as those originating from the recycling of cathode-active materials, appear to be the most suitable for the proposed method. These lithium soaps could also find application in recovery of lithium from dilute aqueous LiCl solutions obtained by washing of loaded aluminum-based adsorbents for *direct lithium extraction* (DLE).^23^

## Data availability

The data supporting this article have been included as part of the ESI.†

## Conflicts of interest

There are no conflicts to declare.

## Supplementary Material

GC-027-D4GC05586A-s001

## References

[cit1] Kim S., Kim J., Kim S., Lee J., Yoon J. (2018). Environ. Sci.: Water Res. Technol..

[cit2] Zhang L., Li L., Rui H., Shi D., Peng X., Ji L., Song X. (2020). J. Hazard. Mater..

[cit3] Srivastava V. , Runtti H., Tuomikoski S., Heponiemi A., Kauppinen T., Tynjälä P. and Lassi U., in Resource Recovery in Industrial Waste Waters, ed. M. Sillanpää, A. Khadir and K. Gurung, Elsevier, Amsterdam, 2023, pp. 545–579

[cit4] Bolan N., Hoang S. A., Tanveer M., Wang L., Bolan S., Sooriyakumar P., Robinson B., Wijesekara H., Wijesooriya M., Keerthanan S., Vithanage M., Markert B., Fränzle S., Wünschmann S., Sarkar B., Vinu A., Kirkham M. B., Siddique K. H. M., Rinklebe J. (2021). Environ. Pollut..

[cit5] Choi H.-B., Ryu J.-S., Shin W.-J., Vigier N. (2019). Nat. Commun..

[cit6] Khalil A., Mohammed S., Hashaikeha R., Hilal N. (2022). Desalination.

[cit7] Nicolaci H. , Young P., Snowdon N., Rai A., Chen T., Zhang J., Lin Y., Bailey E., Shi R. and Zheng N., Direct Lithium Extraction: A Potential Game Changing Technology, Goldman Sachs, New York, 2023

[cit8] Seidell A. and Linke W. F., Solubilities of Inorganic and Metal Organic Compounds: A Compilation of Quantitative Solubility Data from the Periodical Literature, Van Nostrand, New York, 1940

[cit9] Guo X., Cao X., Huang G., Tian Q., Sun H. (2017). J. Environ. Manage..

[cit10] Shin D. J., Joo S., Lee D., Shin S. M. (2022). Can. J. Chem. Eng..

[cit11] Lv Y., Ma B., Liu Y., Wang C., Chen Y. (2024). J. Environ. Chem. Eng..

[cit12] Rosenheim A., Reglin W. (1921). Z. Anorg. Allg. Chem..

[cit13] Stubblefield C. B., Bach R. O. (1972). J. Chem. Eng. Data.

[cit14] Li L. Y. , Chinese Pat, 103964474A, 2014

[cit15] Li L. Y. , Chinese Pat, 104310445A, 2015

[cit16] Laughlin R. G. , in Handbook of Detergents, Part A, ed. G. Broze, CRC Press, Boca Raton, 1999, pp. 99–131

[cit17] Klein R., Touraud D., Kunz W. (2008). Green Chem..

[cit18] McBain J. W., Sierichs W. C. (1948). J. Am. Oil Chem. Soc..

[cit19] Klein R., Tiddy G. J. T., Maurer E., Toraud D., Esquena J., Tache O., Kunz W. (2011). Soft Matter.

[cit20] Hato M., Shinoda K. (1973). J. Phys. Chem..

[cit21] Wan L. S. C., Woon P. K. C. (1969). J. Pharm. Sci..

[cit22] Binnemans K., Jones P. T. (2023). J. Sustainable Metall..

[cit23] Boroumand Y., Razmjou A. (2024). Desalination.

