# Supplementary material for: Removal of lithium from aqueous solutions by precipitation with sodium and choline alkanoate soaps
Source: Green Chem. 2024 Dec 27;27(4):1013–7. doi: 10.1039/d4gc05586a (PMC11702322; doi:10.1039/d4gc05586a)
Supplement: GC-027-D4GC05586A-s001 [file GC-027-D4GC05586A-s001.pdf]

Supplementary Information (SI) for Green Chemistry.
This journal is © The Royal Society of Chemistry 2024

Removal of lithium from aqueous solutions by precipitation with sodium and choline alkanoate soaps

Stijn Raiguel[†], Dženita Avdibegović[†] and Koen Binnemans[†]*

[†] KU Leuven, Department of Chemistry, Celestijnenlaan 200F, P.O. box 2404, B-3001 Leuven, Belgium.

Electronic Supplementary Information (ESI)

*Corresponding author:

Email: Koen.Binnemans@kuleuven.be

Table of contents:

1. Chemicals and reagents S3

2. Instrumentation S3

3. Procedures S4

4. XRD pattern of the C18 precipitate S8

5. Analysis of the reaction mixture above the Krafft point S9

6. ^{7}Li 1D and DOSY NMR S10

7. Titration of choline stearate in the presence of lithium chloride S16

8. Spectra S17

1. Chemicals and reagents:

Myristic acid (99.5%), stearic acid (97%), lithium chloride (99%) and tetrabutylammonium bromide (99+%) were purchased from Acros Organics BV (Geel, Belgium). Dodecanoic acid (98%) was obtained from J&K Scientific BV (Lommel, Belgium). Palmitic Acid (≥99%), chloroform-*d* (99.8 atom% D), 1-butanol (for analysis, Emsure®) and choline hydroxide (46 wt% in water) were procured from Merck Chemicals NV (Hoeilaart, Belgium). Sodium hydroxide (analytical reagent grade), hydrochloric acid (37 wt%), deuterium oxide (for NMR, 99.8 atom% D), Triton X-100 (for electrophoresis) and ethanol (absolute) were purchased from Fischer Scientific BV (Merelbeke, Belgium). Diethyl ether (for HPLC) was obtained from VWR International BV (Leuven, Belgium). All chemicals were used as received, without any further purification. Water was always of ultrapure quality, with a resistivity of 18.2 MΩ·cm and total organic carbon below 5 ppb, provided by a Merck Milli-Q Reference water purification.

2. Instrumentation:

High-field ^{1}H and ^{13}C nuclear magnetic resonance (NMR) spectra were recorded on a Bruker Avance III HD 400 spectrometer with a Bruker Ascend™ 400 magnet system (^{1}H basic frequency of 400.17 MHz) and a 5 mm PABBO BB/19F-1H/D probe with z-gradients. ^{13}C-detected experiments were ^{1}H-decoupled using inverse-gated broadband decoupling. All samples were dissolved in chloroform-*d* ($CDCl_3$). Data were recorded at room temperature using TopSpin 3.7, and processed and analyzed using SpinWorks 4.2.8.0. All data were calibrated using the deuterated solvents as internal calibration reference. The δ-values are expressed in parts per

million (ppm). The following acronyms were used: s (singlet), d (doublet), t (triplet), q (quartet), quint (quintet), m (multiplet).

High-field ^{7}Li NMR spectra were recorded on a Bruker Avance Neo 600 spectrometer with an Ascend™ 600 magnet system and a 5 mm PI HR-TBO (BB/F-H/F-D) probe with z-gradients (max. strength of gradient coil: 53.5 G cm^{-1}). Data were recorded at 80 °C, and recorded, processed and analyzed using TopSpin 4.4.1. Data were calibrated by means of a glass insert containing 1.0 mol L^{-1} LiCl in D_2O (lock reference).

Ion chromatography was carried out on a Shimadzu device consisting of a of a CBM-40 system controller, DGU-403 degassing unit, LC-20Ai pump, SIL20A autosampler, CTO-40C column oven and CDD-10AVP conductivity detector. A Metrohm Metrosep C4 column was used. The eluent consisted of 1.7 mmol L^{-1} nitric acid and 0.7 mmol L^{-1} pyridine-2,6-dicarboxylic acid in ultrapure water. The oven temperature was set at 25°C and the flow rate of the eluent was 0.9 mL min^{-1}. Samples were diluted using ultrapure water. The injection volume was varied between 3 and 10 µL in order to obtain both sufficient peak intensity and resolution. Calibration was performed using an external standard solution series, to which a linear calibration curve was fitted and forced through zero in order to obtain more accurate quantification for highly dilute samples.

Infrared spectra were collected using a Bruker Vertex 70 spectrometer equipped with Platinum ATR module, and analyzed using the Opus 7 Software package.

XRD diffractograms were collected on a Bruker D2 Phaser 2nd Generation, with a goniometer radius of 121 nm and a Cu Kα/β X-ray source with wavelengths of 1.54060 and 1.54439 Å,

respectively. Data were recorded for values of 2θ between 5 and 90 °, with a step size of 0.05 ° and a measurement time if 1 second per step.

3. Procedures

Synthesis of sodium soaps

A 20 mL aliquot of a 2 mol L^{-1} solution of NaOH in ethanol (40 mmol, 11 eq.) was combined with 3.64 mmol of the fatty acid (728 mg of lauric acid, 830 mg of myristic acid, 932 mg of palmitic acid or 1034 mg of stearic acid) in a 20 mL vial. The mixture was stirred at ambient temperature (22 °C) for 2 hours and at 60 °C overnight. The resultant cake was broken up, suspended in 30 mL of ethanol and filtered. The residue after filtration was washed three times with 25 mL of ethanol, until the filtrate had a pH of 7. The cake was dried overnight at 60 °C to afford a light pink (myristate, palmitate and stearate) or light orange (laurate) solid.

Yield:

Sodium laurate: 69% (561 mg, 2.52 mmol)

FT-IR (υ/cm^{-1}): 2953, 2922, 2848 (C-H stretching), 1556 (carboxylate asymmetrical C-O stretching), 1462 (C-H bending), 1444, 1422 (carboxylate symmetrical C-O stretching), 923, 724, 697.

Sodium myristate: 89% (812 mg, 3.24 mmol)

FT-IR (υ/cm^{-1}): 2953, 2920, 2847 (C-H stretching), 1557 (carboxylate asymmetrical C-O stretching), 1462 (C-H bending), 1445, 1422 (carboxylate symmetrical C-O stretching), 923, 723, 698.

Sodium palmitate: 92% (932 mg, 3.35 mmol)

FT-IR (υ/cm^{-1}): 2953, 2918, 2847 (C-H stretching), 1556 (carboxylate asymmetrical C-O stretching), 1462 (C-H bending), 1445, 1420 (carboxylate symmetrical C-O stretching), 923, 723, 698.

Sodium stearate: 95% (1056 mg, 3.45 mmol)

FT-IR (υ/cm^{-1}): 2954, 2916, 2873, 2848 (C-H stretching), 1556 (carboxylate asymmetrical C-O stretching), 1463 (C-H bending), 1441, 1419 (carboxylate symmetrical C-O stretching), 923, 879, 719, 698.

Infrared analysis demonstrated full conversion of the fatty acid to the soap (full spectra shown in Section 4).

Synthesis of choline soaps

A 1 mL aliquot of choline hydroxide solution (4.1 mmol) and 10 mL of water were combined with 3.5 mmol of fatty acid (897.5 mg of palmitic acid or 955.7 mg of stearic acid). In the synthesis of choline palmitate, the sample was magnetically stirred at ambient temperature for 1 hour. In the synthesis of choline stearate, the sample was stirred at 45 °C for 1 hour. The resulting emulsion was transferred to a flask and 10 mL of 1-butanol was added (anti-foaming agent) prior to the removal of solvents and decomposition of excess choline hydroxide under reduced pressure at 60 °C.

The residue was washed twice with 10 mL of diethyl ether. The product was then allowed to air dry for 3 days. Finally, traces of water were removed by lyophilization. The product collected as a white (palmitate) or off-white (stearate) waxy powder.

Yield:

Choline palmitate: 58% (734.7 mg, 2.043 mmol)

^{1}H NMR ($CDCl_3$, 400 MHz, δ/ppm): 4.17 (2 H, broad m, C**H**$_2$-OH), 3.79 (2H, m, C**H**$_2$-N^+), 3.39 (9H, s, N-C**H**$_3$), 2.19 (2H, t, ^3J = 8.0 Hz, C**H**$_2$-COO^-), 1.62 (2H, 5-et, ^3J = 7.5 Hz, C**H**$_2$- CH_2-COO^-), 1.26 (24H, m, 12 C**H**$_2$), 0.90 (3H, t, ^3J = 7.0 Hz, C**H**$_3$).

^{13}C NMR ($CDCl_3$, 100 MHz, δ/ppm): 180.59 (**C**OO^-), 68.85 (**C**H_2-OH), 56.06 (**C**H_2-N^+), 54.83 (N-**C**H_3), 39.21 (**C**H_2-COO^-), 31.94 (C**H**$_2$), 30.06 (C**H**$_2$), 29.72 (8 C**H**$_2$), 29.67 (C**H**$_2$), 29.37 (C**H**$_2$), 27.16 (C**H**$_2$), 22.70 (C**H**$_2$), 14.13 (C**H**$_2$).

Choline stearate: 59% (802.7 mg, 2.071 mmol)

^{1}H NMR ($CDCl_3$, 400 MHz, δ/ppm): 4.17 (2 H, broad m, C**H**$_2$-OH), 3.79 (2H, m, C**H**$_2$-N^+), 3.39 (9H, s, N-C**H**$_3$), 2.19 (2H, t, ^3J = 8.0 Hz, C**H**$_2$-COO^-), 1.62 (2H, quint, ^3J = 7.5 Hz, C**H**$_2$- CH_2-COO^-), 1.27 (28H, m, 12 C**H**$_2$), 0.90 (3H, t, ^3J = 7.0 Hz, C**H**$_3$).

^{13}C NMR ($CDCl_3$, 100 MHz, δ/ppm): 180.58 (**C**OO^-), 68.83 (**C**H_2-OH), 56.05 (**C**H_2-N^+), 54.82 (N-**C**H_3), 39.23 (**C**H_2-COO^-), 31.94 (C**H**$_2$), 31.94 (C**H**$_2$), 30.07 (C**H**$_2$), 29.72 (10 C**H**$_2$), 29.67 (C**H**$_2$), 29.37 (C**H**$_2$), 27.17 (C**H**$_2$), 22.70 (C**H**$_2$), 14.13 (C**H**$_2$).

Precipitation

A 20 mL aliquot of the lithium chloride feed solution was transferred to a 20 mL vial charged with 1.05 equivalents of the precipitating agent with respect to lithium (unless stated otherwise). The sample was stirred at 500 rpm for 30 minutes (unless stated otherwise) at a temperature of 5

°C above the Krafft point, unless the Krafft point was below room temperature, in which case it was stirred at ambient temperature (22 °C). The solution was then allowed to cool and settle. A sample was taken and filtered over a 0.45 µm syringe filter and diluted for analysis using ion chromatography. If multiple replicates were measured, errors are reported as the standard error on the mean.

4. XRD Pattern of the C18 precipitate

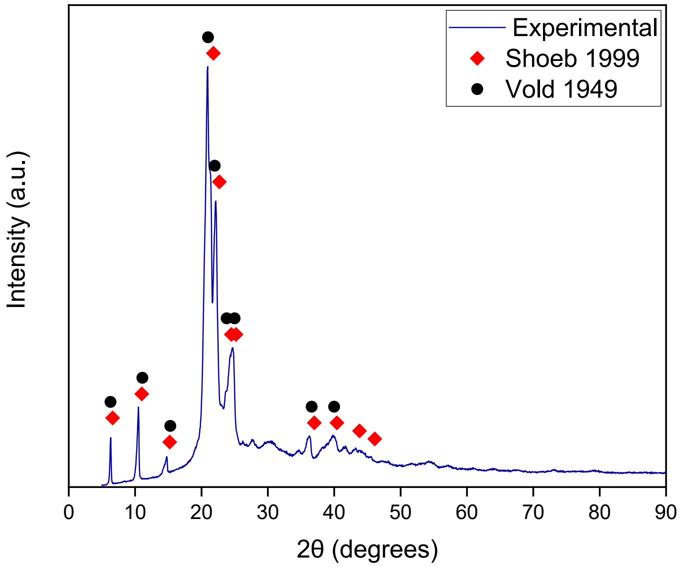

Fig. S1: XRD diffractogram of the precipitate obtained by treatment of LiCl solution with choline stearate, compared to two literature patterns for lithium stearate.[1,2]

5. Analysis of the reaction mixture above the Krafft point

In order to determine the lithium concentration in the reaction mixture above the Krafft point, care must be taken to prevent the formation of precipitates upon cooling during sample preparation. To this end, a modified sample preparation procedure was carried out wherein the samples were diluted using a 2 vol% Triton X-100 solution in ultrapure water prior to analysis using IC. This sufficiently depresses the Krafft point of sodium stearate to prevent precipitation at room temperature.

Samples of the reaction mixture above the Krafft point was prepared by solutions containing 250 ppm of lithium (as LiCl) and various concentrations of NaCl with 110.5 mg (1.05 eq.) of sodium stearate, and stirring for overnight at 80 °C. The mixtures were then rapidly filtered over a syringe filter with pore size 0.45 µm without cooling. Subsequently, the filtered solution was diluted for analysis while maintaining the temperature at 80 °C. The effect of volume changes of the sampled aliquots upon cooling was taken into account when calculating the final dilution factors. The equilibrium lithium concentration was found to increase with increasing NaCl concentrations: 27.8 ppm Li for 5 g L^{-1} NaCl, 107.3 ppm for 30 g L^{-1} NaCl and 174.5 ppm for 50 g L^{-1} NaCl.

6. ^{7}Li 1D and DOSY NMR

One-dimensional ^{7}Li NMR spectra were recorded at 80 °C of a sample containing free, solvated Li^{+} ions in water (1), and a sample representative of the solution at equilibrium during the metathesis process (2). These were represented by: (1) LiCl (500 ppm Li) in ultrapure water, and (2) the liquid phase of a reaction mixture between LiCl (500 ppm Li) and 221 mg (1.05 eq.) of sodium stearate in ultrapure water. The latter sample was prepared by heating the reactants to 80 °C and filtering immediately (without cooling) using a syringe filter with 0.45 µm pore size. A zg30 pulse program was applied, with a recycle delay time of 2 s and acquisition time of 5.26 s

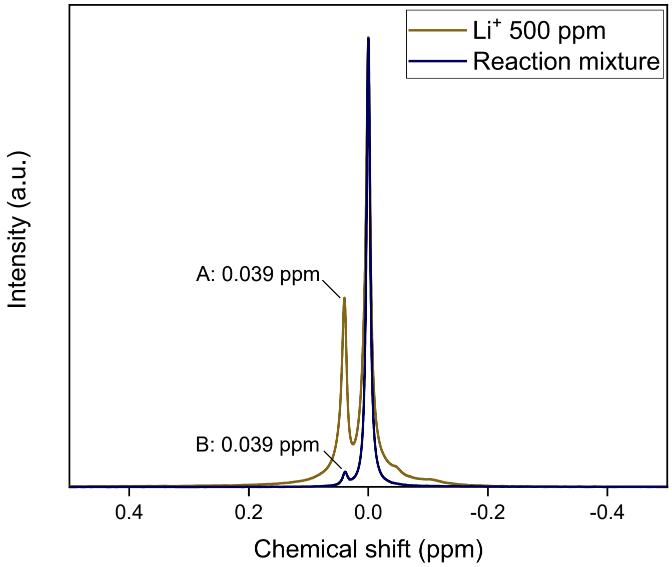

Fig. S2: 1D ^{7}Li NMR spectra of A) 500 ppm Li^{+} as LiCl in water and B) reaction mixture containing 500 ppm Li^{+} as LiCl and 1.05 eq. of sodium stearate in water, after filtration at 80 °C. Both spectra were recorded at 80 °C and referenced to 1.00 mol L^{-1} LiCl in D_2O (0.00 ppm).

As can be seen from the spectra shown in Fig. S2, the chemical shift of ^{7}Li was nearly identical in both samples at 0.039 ppm, indicating that the immediate chemical environment of Li is not significantly altered by the presence of micelles formed by the excess of sodium stearate. In other words, it is unlikely that stearate monomers or micelles are present in the inner solvation shells of the Li^+ ion.

In a follow-up experiment, ^{7}Li diffusion-ordered spectroscopy (DOSY) measurements were performed on the same two samples at 80 °C. The reference capillary containing 1.00 mol L^{-1} LiCl in D_2O was replaced by one containing only D_2O, providing a lock reference without the risk of overlap between ^{7}Li nuclei in the sample and those in the reference capillary. A ledbpgp2s pulse program was applied with varying gradient strength. The parameters were used are given in Table S1.

Table S1: Values used for experimental parameters in ^{7}Li DOSY NMR experiments.

	**LiCl (500 ppm Li)**	**Reaction mixture (measurement 1)**	**Reaction mixture (measurement 2)**
Gradient length (δ, ms)	2.6	2.6	2.6
Gradient strength (g, G cm^{-1})	2.0-50.5	2.0-50.5	2.0-50.5
Gradient ramp	Linear, 16 steps	Linear, 16 steps	Linear, 16 steps
Diffusion time (Δ, ms)	59.9	19.9	39.9

The experimentally recorded signal intensities (calculated as area integrals) were fitted to a function of the form (Eq. S1):

$$I(g) = I_0 e^{-D\gamma^2 g^2 \delta^2 (\Delta - \frac{\delta}{3})} \tag{S1}$$

Wherein I_0 denotes the reference intensity (unattenuated intensity at zero gradient strength), D represents the diffusivity of the observed species and γ is the gyromagnetic ratio of the nucleus being recorded, in this case ^{7}Li (γ = 10397 rad s^{-1} G^{-1}).

These fits are shown in Figs. S3, S4 and S5. The values for D obtained from the fit are (3.0 ± 0.1) 10^{-9} m^2 s^{-1} for the Li^+ ion in the 500 ppm solution, (2.5 ± 0.2) 10^{-9} m^2 s^{-1} for the Li^+ ion in the reaction mixture with Δ = 19.9 ms and (2.7 ± 0.2) 10^{-9} m^2 s^{-1} for the Li^+ ion in the reaction mixture with Δ = 39.9 ms. The errors on these numbers are given as the errors on the estimates of D as obtained by regression of eq. S1 to the experimental datasets, with a confidence level of 95%. The values obtained for the reaction mixture have a larger error due to a lower signal-to-noise ratio of the spectra. The data collected during measurement conducted with Δ = 19.9 ms has a higher signal-to-noise ratio than that with Δ = 39.9 ms (due to more scans being collected), but, as the diffusion time was insufficient for the signal to fully decay at maximal gradient strength (see Fig. S4), Eq. S1 was only fitted over a limited range of values of *I(g)*. As a result, both measurements yield roughly equally precise estimates of *D*. These estimates do not differ significantly from each other at the given confidence level. The measured value of *D* for aqueous

LiCl differs significantly (CL = 95%) from *D* that of Li in the reaction mixture obtained with Δ = 19.9 ms, but not from that obtained with Δ = 39.9 ms. Presumably, this is due to the uncertainty resulting from the low signal-to-noise ratio for the data collected during the latter measurement. Taken together, however, these data indicate that there is a small but significant retardation of the diffusion of Li^+ in the presence of the 5 mol% excess of sodium stearate present in the reaction mixture.

In conclusion, the ^7Li NMR measurements show that the immediate solvation environment of Li^+ is not significantly different in the reaction mixture as compared to a dilute solution of LiCl. There is, however, a small reduction in the diffusivity of Li^+, but it is of a smaller magnitude than could be expected if Li^+ was firmly adsorbed to the surface of a micelle, as micelles have diffusion constants which are orders of magnitude lower than those of free ions.[3,4] It thus appears that the Li+ ions are present in the outer, diffuse adsorption layer of the micelles (where exchange is comparatively rapid), rather than the inner stern adsorption layer.[5]

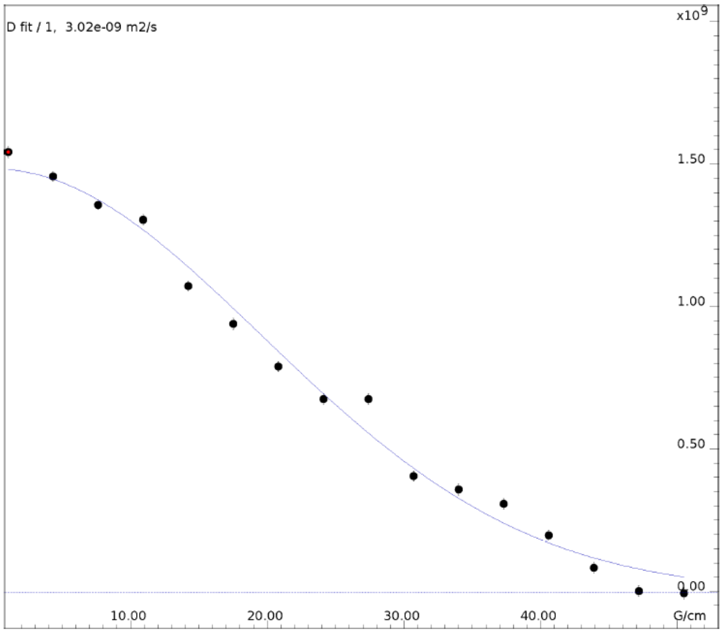

Fig. S3: ^{7}Li signal intensity I(g) as a function of the gradient strength g for 500 ppm Li^{+} as LiCl in water at 80 °C. Experimental parameters as given in Table S1. Error bars correspond to the RMS of the signal intensity in each individual spectrum.

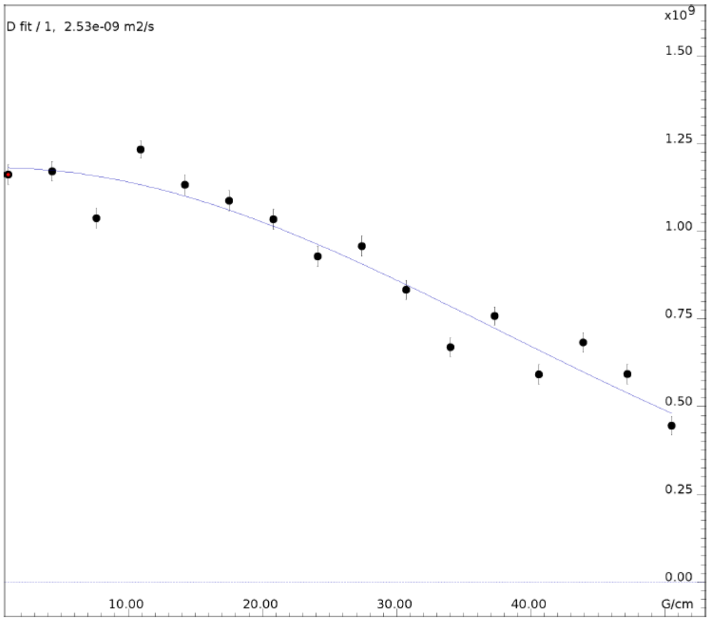

Fig. S4: ^{7}Li signal intensity I(g) as a function of the gradient strength g for the reaction mixture resulting from 500 ppm Li^{+} as LiCl and 1.05 eq. sodium stearate in water at 80 °C, with Δ = 19.9 ms. Experimental parameters as given in Table S1. Error bars correspond to the RMS of the signal intensity in each individual spectrum.

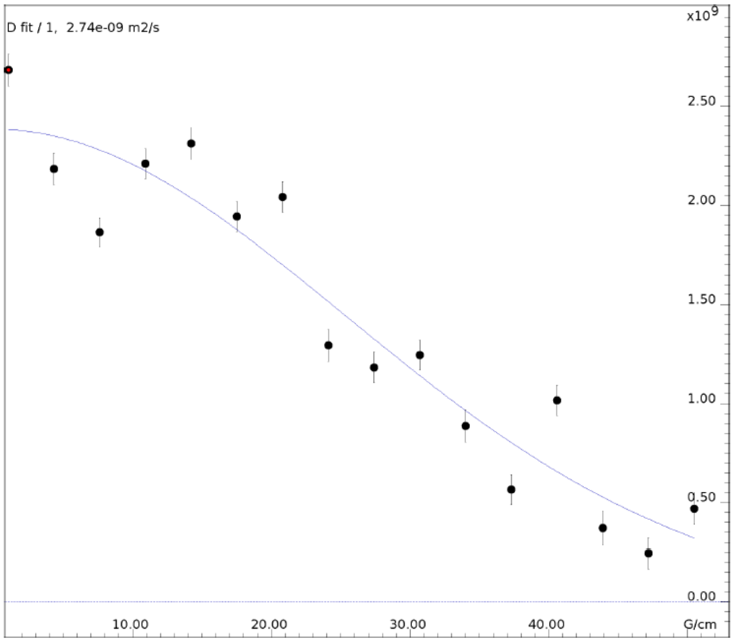

Fig. S5: ^{7}Li signal intensity I(g) as a function of the gradient strength g for the reaction mixture resulting from 500 ppm Li^{+} as LiCl and 1.05 eq. sodium stearate in water at 80 °C, with Δ = 39.9 ms. Experimental parameters as given in Table S1. Error bars correspond to the RMS of the signal intensity in each individual spectrum.

7. Titration of choline stearate in the presence of lithium chloride

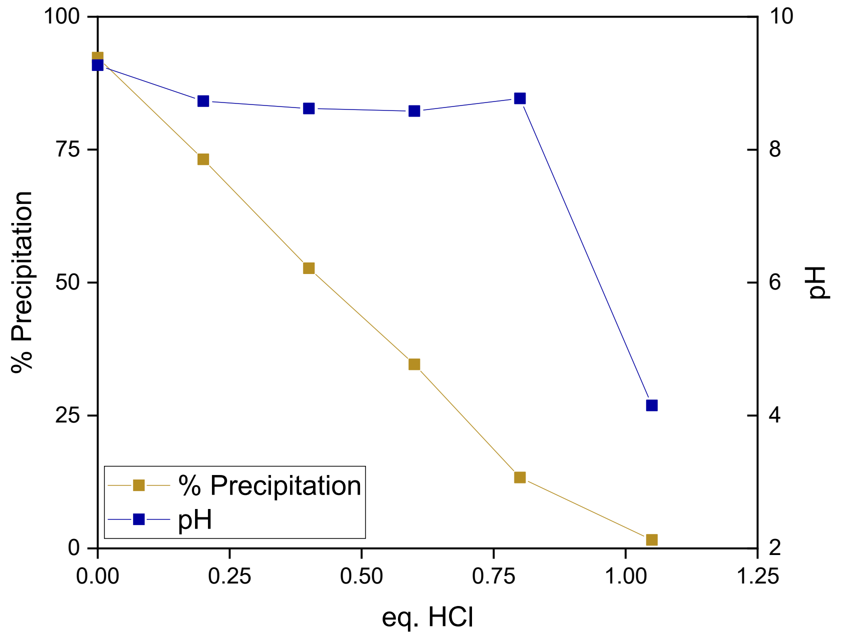

Fig. S6: Precipitation yield (%) and pH during the titration of choline palmitate (1.05 eq.) with hydrochloric acid in the presence of 91 ppm lithium (as LiCl).

8. Spectra

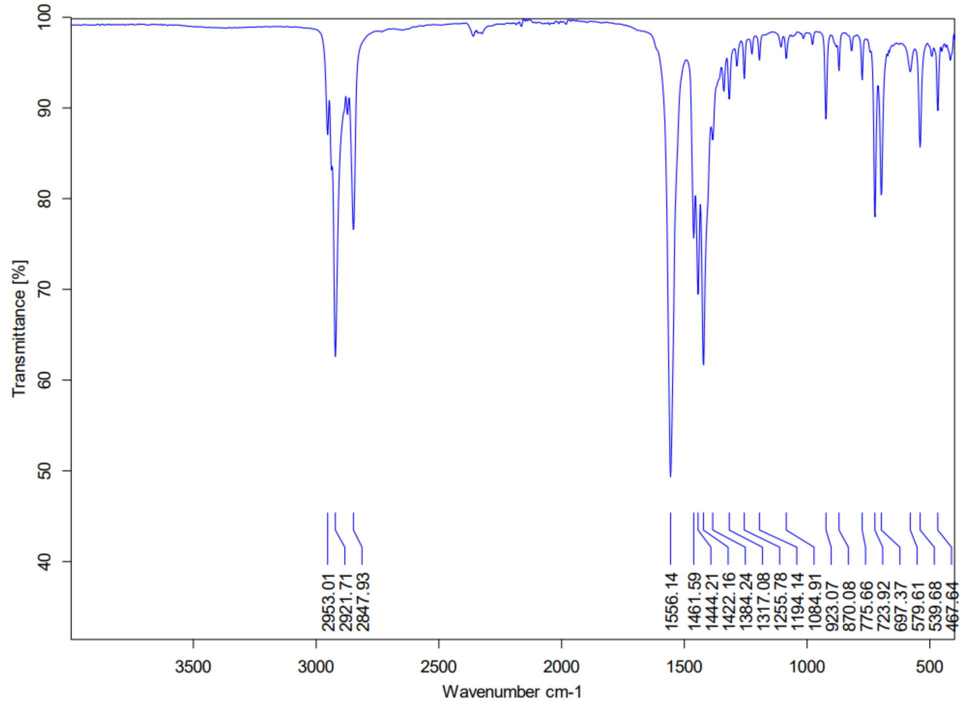

Fig. S7: FT-IR spectrum of sodium laurate.

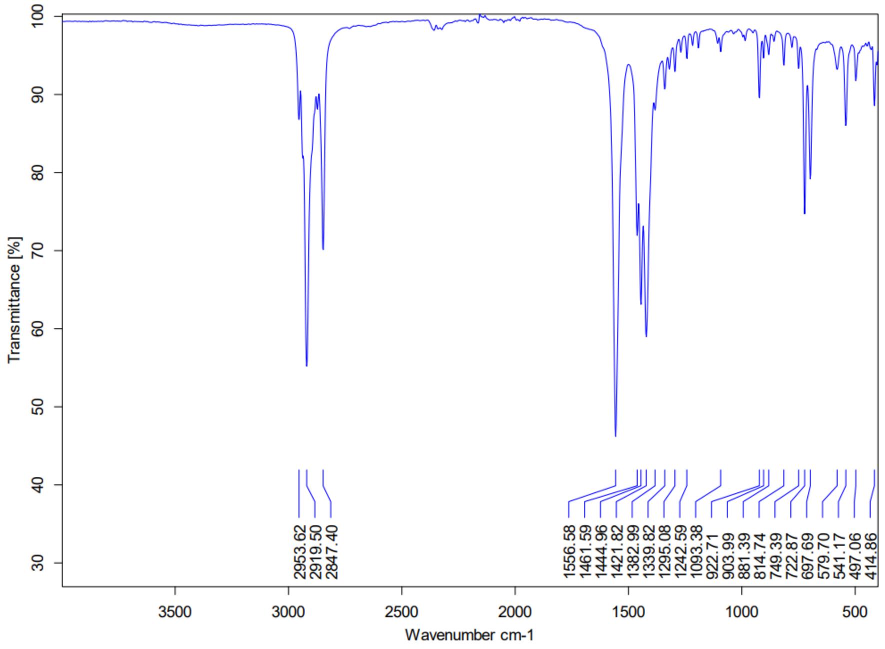

Fig. S8: FT-IR spectrum of sodium myristate.

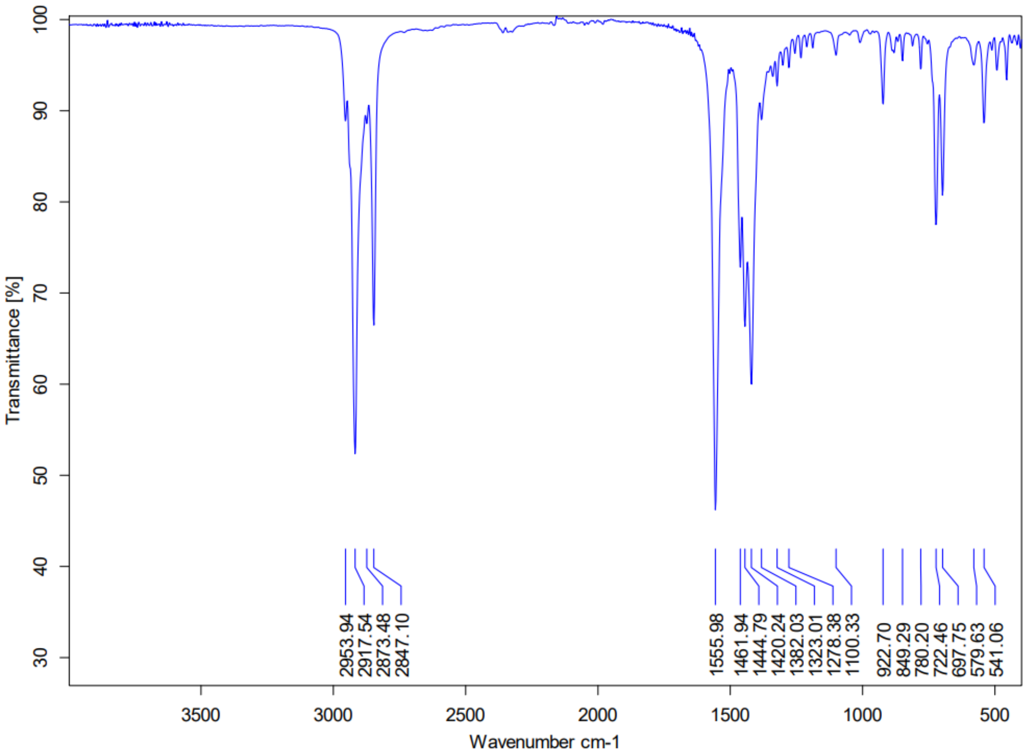

Fig. S9: FT-IR spectrum of sodium palmitate.

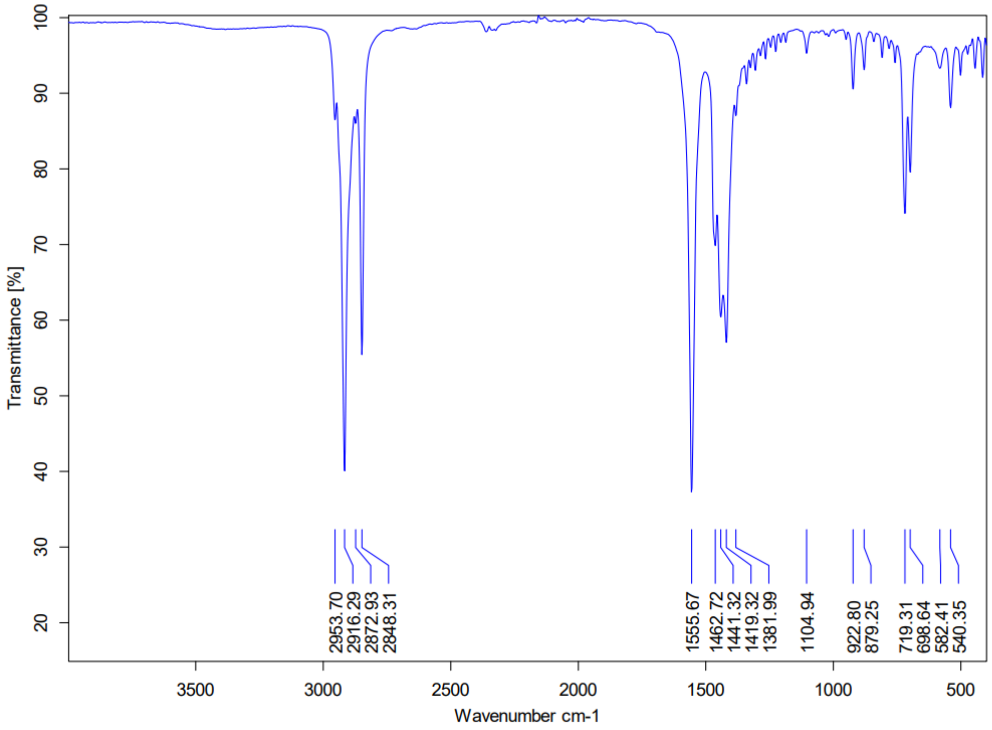

Fig. S10: FT-IR spectrum of sodium stearate.

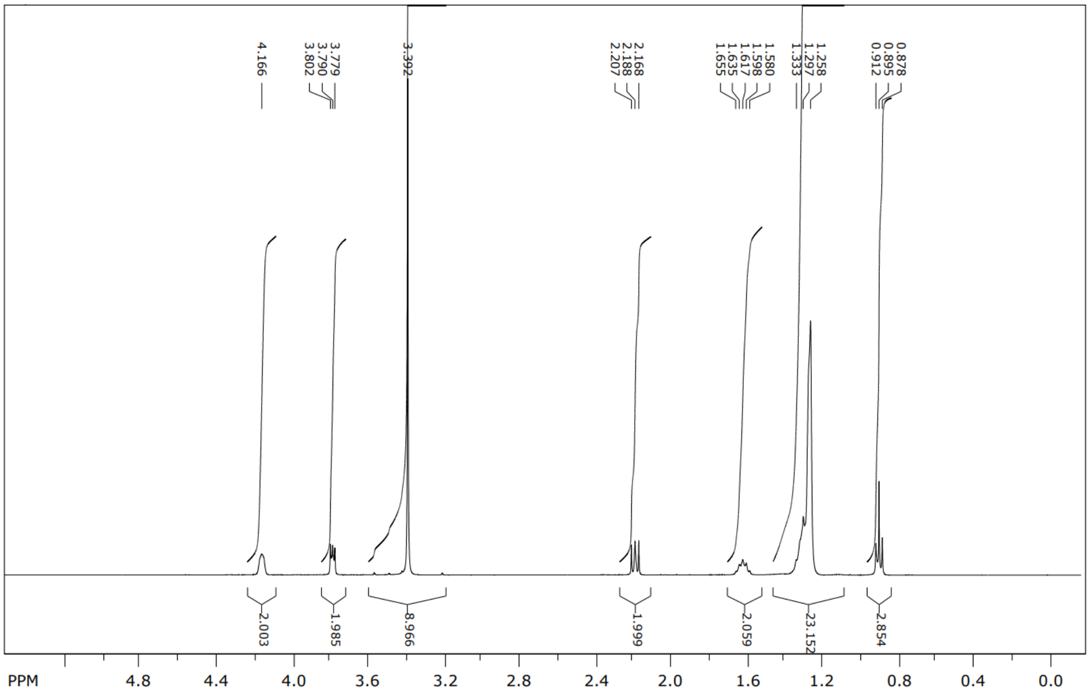

Fig. S11: ^{1}H NMR spectrum of choline palmitate.

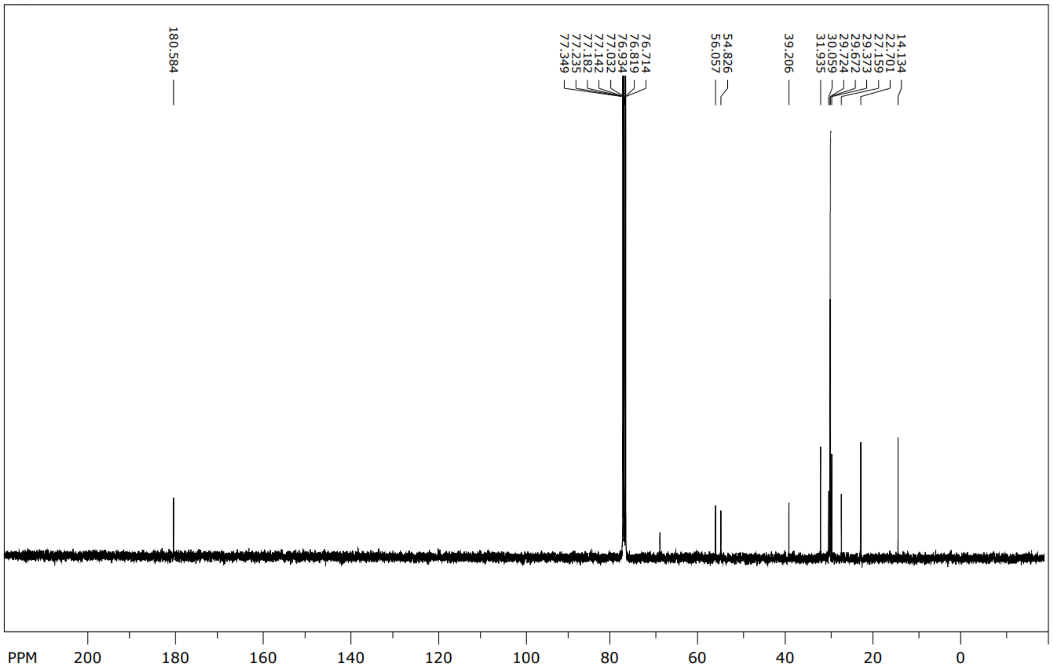

Fig. S12: ^{13}C NMR spectrum of choline palmitate.

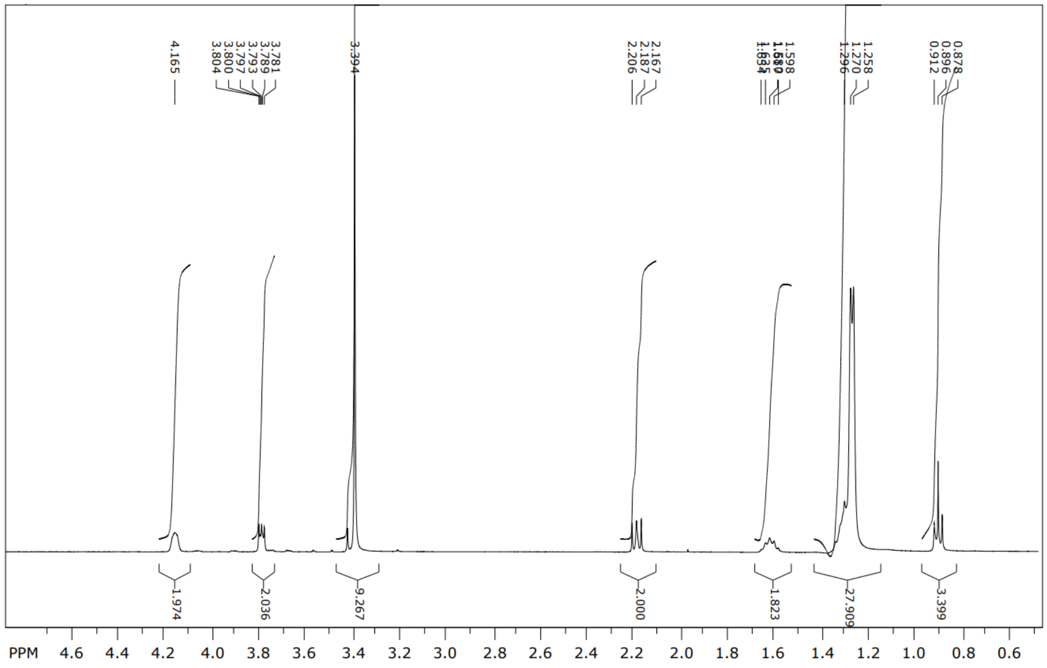

Fig. S13: ^{1}H NMR spectrum of choline stearate

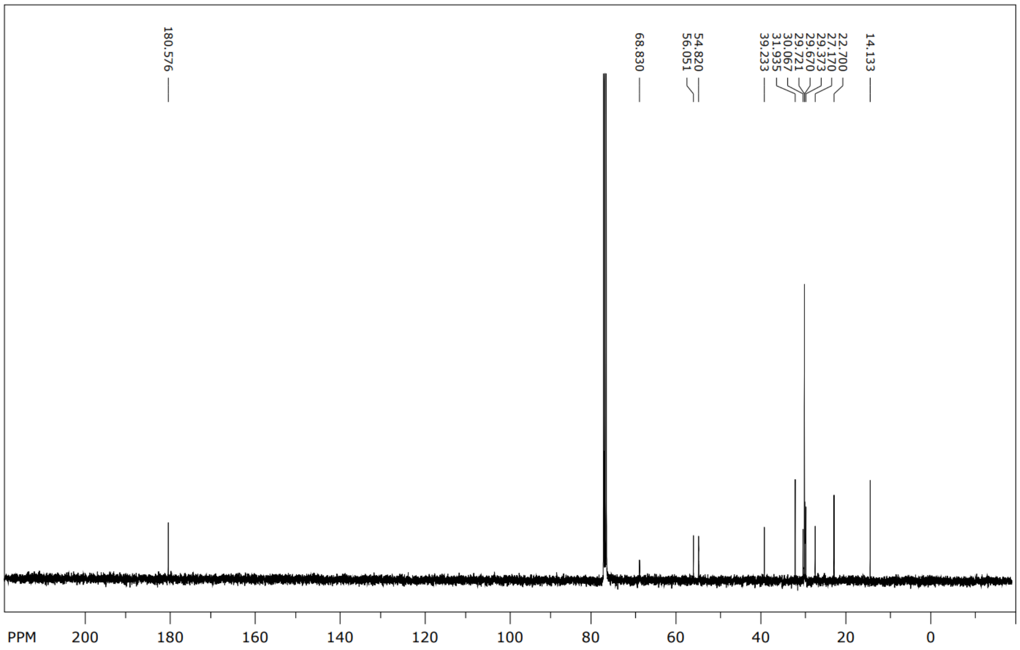

Fig. S14: ^{13}C NMR spectrum of choline stearate.

9. References

1 Z. E. Shoeb, B. M. Hammad and A. A. Yousef, *Grasas Aceites*, 1999, **50**, 426–434.

2 M. J. Vold and R. D. Vold, *J. Colloid Sci.*, 1950, **5**, 1–19.

3 T. G. Movchan, A. I. Rusanov, I. V. Soboleva, N. R. Khlebunova, E. V. Plotnikova and A. K. Shchekin, *Colloid J*, 2015, **77**, 492–499.

4 F. Luschtinetz and C. Dosche, *J. Colloid Interface Sci.*, 2009, **338**, 312–315.

5 K. D. Danov, S. D. Kralchevska, P. A. Kralchevsky, G. Broze and A. Mehreteab, *Langmuir*, 2003, **19**, 5019–5030.
